# Enhanced antitumor effect of biodegradable cationic heparin-polyethyleneimine nanogels delivering FILIP1LΔC103 gene combined with low-dose cisplatin on ovarian cancer

**DOI:** 10.18632/oncotarget.19464

**Published:** 2017-07-22

**Authors:** Chuan Xie, Maling Gou, Tao Yi, Xiaorong Qi, Ping Liu, Yuquan Wei, Xia Zhao

**Affiliations:** ^1^ Department of Gynecology and Obstetrics, Key Laboratory of Birth Defects and Related Diseases of Women and Children of The Ministry of Education, West China Second Hospital, Sichuan University, Chengdu, Sichuan 610041, P.R. China; ^2^ State Key Laboratory of Biotherapy and Cancer Center, West China Hospital, Sichuan University, Chengdu 610041, P.R. China

**Keywords:** filamin a interacting protein 1-like, cisplatin, heparin-polyethyleneimine, gene therapy, ovarian cancer

## Abstract

FILIP1LΔC103 (COOH terminal truncation mutant 1-790 of Filamin A Interacting Protein 1-Like) has been identified to hold therapeutic potential for suppressing tumor growth. Cisplatin (DDP) is commonly used as a first-line drug in the treatment for ovarian cancer. The usage of polymeric nanoparticles to deliver functional genes intraperitoneally holds much promise as an effective therapy for ovarian cancer. In this study, a recombinant plasmid expressing FILIP1LΔC103 (FILIP1LΔC103-p) was constructed, and HPEI nanogels were prepared to deliver FILIP1LΔC103-p into SKOV3 cells. The expression of FILIP1LΔC103 *in vitro* and *in vivo* was determined using RT-PCR and Western Blotting. Moreover, *in vivo* treatment experiments were conducted on nude mice bearing SKOV3 ovarian cancer. The mice were treated with 5% glucose, HPEI+E-p, HPEI+FILIP1LΔC103-p, DDP or HPEI+FILIP1LΔC103-p plus DDP, respectively. Tumor weights were evaluated throughout the treatment duration. The cell proliferation and apoptosis were evaluated by Ki-67 immunochemical staining and TUNEL assay respectively, and the anti-angiogenic effect was assessed by CD31 immunochemical staining and alginate-encapsulated tumor cell assay. FILIP1LΔC103-p could be efficiently transfected into SKOV3 cells by HPEI nanogels. The combination of HPEI+FILIP1LΔC103-p with DDP exerted enhanced antitumor activity compared with HPEI+FILIP1LΔC103-p or DDP alone. Significant reduction of tumor cells proliferation, augmentation of tumor cells apoptosis and suppression of angiogenesis were observed in the combination group compared with controls. Our results demonstrated synergistic antineoplastic activity of combined FILIP1LΔC103 and low-dose DDP with no apparent toxicity, indicating a potential application of the combined approach in the treatment of ovarian cancer.

## INTRODUCTION

FILIP1LΔC103 (COOH terminal truncation mutant 1-790 of Filamin A Interacting Protein 1-Like) was recognized as a prospectively significant regulator of angiogenesis activity. Up-regulation of FILIP1LΔC103 expression can lead to the induction of apoptosis of tumor cell and inhibition of proliferation of tumor cell [1]. Moreover, we previously found that targeted expression of FILIP1LΔC103 in tumor cells could effectively inhibit tumor growth *in vivo* [2]. A recent study has shown that reduced expression of FILIP1L in ovarian cancer is related to the invasive phenotype [3]. Based on these findings, FILIP1LΔC103, as a significant regulator of tumor cell migration and growth, has the prospective to be a target for gene therapy in ovarian cancer.

Ovarian cancer, which accounts for most of the deaths from all gynecologic malignancies, is one of the major female health problems all over the world, and it ranks fifth in causing female death due to cancer [4]. Most patients are diagnosed at advanced stage, and currently, the standard management for advanced ovarian cancer is a combination of surgery and chemotherapy [5]. Cisplatin (DDP) is a first-line chemotherapeutic agent in the therapy for ovarian cancer. The DDP's antitumor mechanisms include failing to repair damaged DNA, building bifunctional DNA crosslinks, interference with replication of DNA and induction of cell apoptosis and necrosis [6–8]. Despite the significant effect on ovarian carcinoma, DDP still has some disadvantages clinically, including drug resistance and severe side effects. In addition, the gynecologic oncologists have reached a plateau with these conventional chemotherapeutic agents. In such a situation, an aggressive chemotherapy may not only affect the quality of life but also may not be beneficial to the patients’ survival. To overcome these hurdles for the successful use of DDP, effective and novel therapeutic strategies, such as gene therapy combined with chemotherapy as well as increasing therapeutic effects of chemotherapy, are urgently needed to fight against ovarian cancer. Based on these factors, this study seeks to develop combination therapy of FILIP1L and low-dose DDP for therapy of ovarian carcinoma, with the intention of evaluating its potential effect against ovarian carcinoma.

The therapeutic approach of targeted gene therapy's application for cancer has been recognized broadly, however, its clinical usage is limited mainly because of failure to find a competent and safe gene delivery system. Currently, cationic PEI (polyethyleneimine) is one of the most effective non-viral gene carriers for targeted gene transfer [9–11]. However, PEI is unbiodegradable and its application is greatly restricted because of its transfection efficiency, chain length, and correlation of cytotoxicity. A way to address the issues is to couple the PEI chains using a biodegradable linker to form a longer chain. In our laboratory, biodegradable cationic nanogels HPEI (heparin polyethyleneimine) are synthesized when low molecular weight PEI2K is chemically conjugated by heparin [2, 12], and the HPEI nanogels are deployed as a non-viral carrier for gene transfer in this study.

This study demonstrates that FILIP1LΔC103 could effectively inhibit tumor growth when over-expressed in cancer cells, and the antitumor efficacy of FILIP1LΔC103 was enhanced when combined with low-dose DDP without an overt increase in toxicity. In addition, the result also shows that HPEI nanogels can efficiently deliver a plasmid expressing FILIP1LΔC103 into ovarian carcinoma cells *in vivo* and in-vitro.

## RESULTS

### Preparation and physical properties of HPEI nanogels

Catalyzed by NHS and EDC, HPEI are obtained when PEI2K is chemically coupled by heparin. The HPEI nanogels enclosing FILIP1LΔC103 are monodispersed and appeared as a 28 nm diameter sphere in the transmission electron microscopy imaging.

### Expression of FILIP1LΔC103 *in vitro*

**Figure 1:**
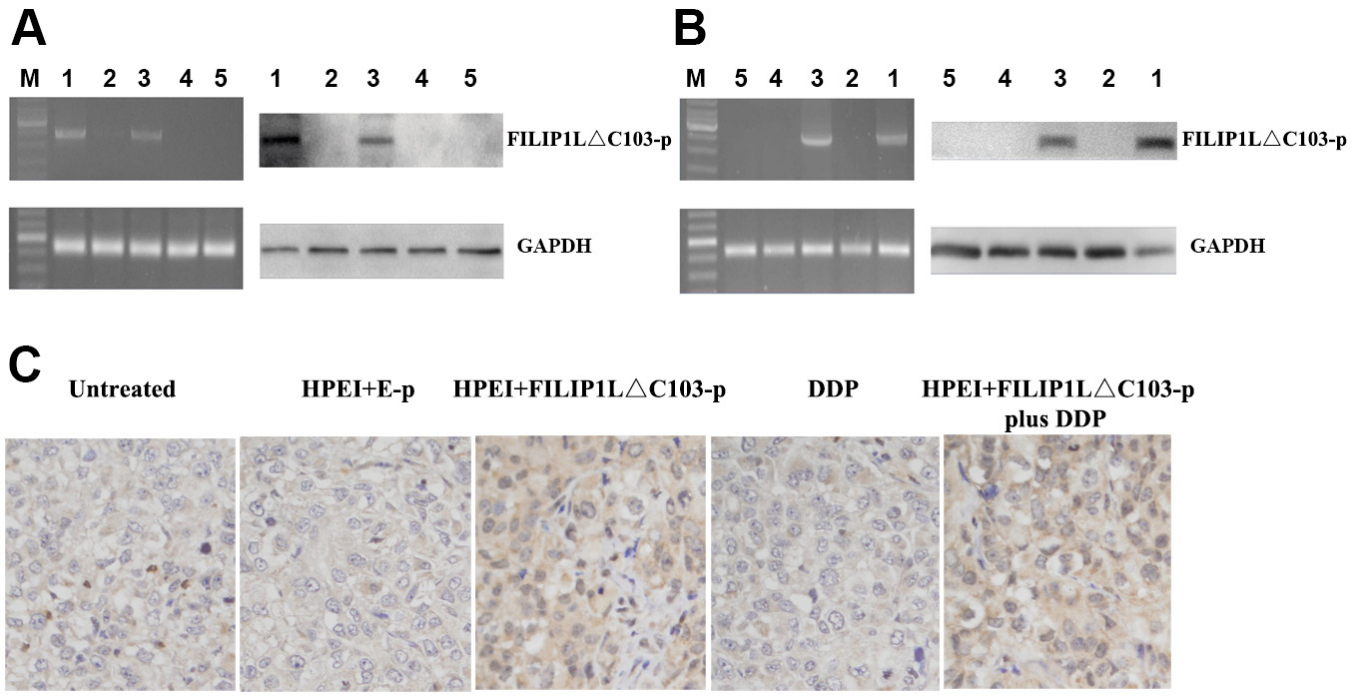
Expression of FILIP1LΔC103-p *in vitro* and *in vivo* **(A)** identification of the expression of FILIP1LΔC103-p in transfected SKOV3 cells *in vitro* by RT-PCR and Western Blot analysis. SKOV3 ovarian cancer cells were treated with HPEI+FILIP1LΔC103-p plus DDP (1), DDP (2), HPEI+FILIP1LΔC103-p complexes (3), HPEI+E-p complexes (4), or 5% GS (5) respectively. On the left, RT-PCR indicated only HPEI+FILIP1LΔC103-p complexes treated cells(Lane 1 and 3) had a positive band of FILIP1LΔC103-p (2370 bp). GAPDH was used as the internal standard. On the right, the result of Western Blot showed positive band (~100 kDa) only occurring in cells transfected with HPEI+FILIP1LΔC103-p complexes in contrast to control groups. **(B)** Expression of FILIP1LΔC103 *in vivo*. The intraperitoneal carcinomatosis model in nude mice was established and intraperitoneally administered with HPEI+FILIP1LΔC103-p plus DDP (1), DDP (2), HPEI+FILIP1LΔC103-p complexes (3), HPEI+E-p complexes (4), or 5% GS (5) respectively. **(C)** Immuno-staining of FILIP1LΔC103 in tumor tissue, original magnification ×400.

To detect whether the HPEI+FILIP1LΔC103-p complexes could lead to the expression of FILIP1LΔC103 *in vitro*, ovarian cancer cells SKOV3 were treated with 5% glucose, HPEI+E-p complexes, HPEI+FILIP1LΔC103-p complexes, DDP and HPEI+FILIP1LΔC103-p complexes plus DDP, respectively. FILIP1LΔC103’ expression was discovered when RT-PCR and Western Blotting was carried out after transfection for 48 hours. The HPEI nanogels could effectively transfect FILIP1LΔC103 into SKOV3 cells *in vitro*. FILIP1LΔC103 could be re-expressed in SKOV3 ovarian cancer cells after transfection with HPEI+FILIP1LΔC103-p complexes, when the three control groups (Figure 1A) are compared.

### Expression of FILIP1LΔC103 *in vivo*

A human ovarian cancer intraperitoneal xeno-graft model was established to examine whether the HPEI+FILIP1LΔC103-p complexes resulted in expression of FILIP1LΔC103 *in vivo*. The above five agents were respectively injected into mice. All the nude mice were euthanized after the last 3 days of injection and the tumor nodules from the nude mice were extracted for RT-PCR and Western Blotting. The HPEI+FILIP1LΔC103-p could lead to expression of FILIP1LΔC103 *in vivo*, while no FILIP1LΔC103 expression could be observed in the other three control groups which were untreated with HPEI+FILIP1LΔC103-p (Figure 1B)

### Inhibition of ovarian cancer by combination therapy

**Figure 2:**
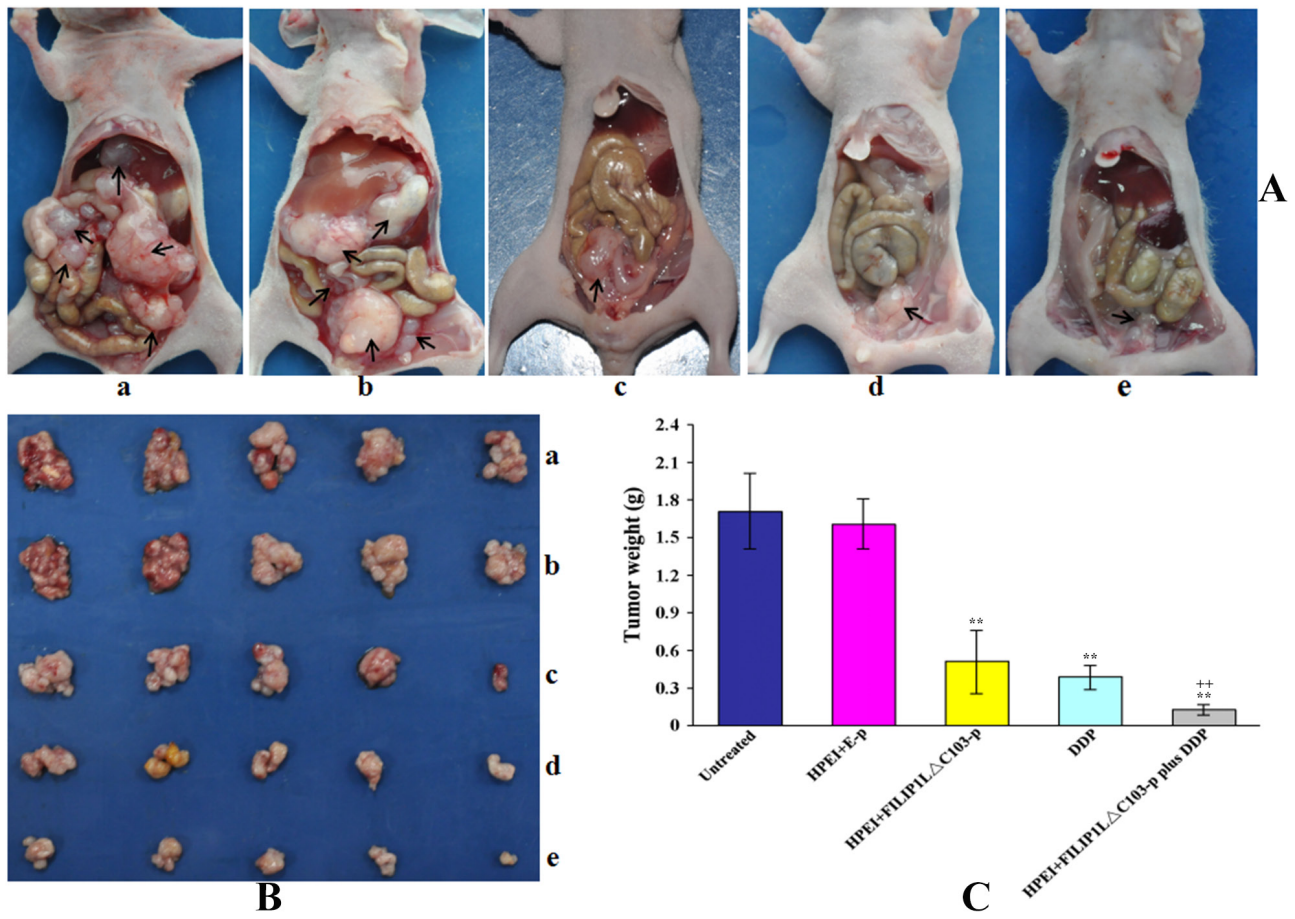
Tumor weights in the intraperitoneal xenograft model of human ovarian cancer in nude mice The mice were intraperitoneally administered with 5%GS (a), HPEI+E-p complexes (b), HPEI+FILIP1LΔC103-p complexes (c), DDP (d) or HPEI+FILIP1LΔC103-p plus DDP (e), respectively. The result indicated that FILIP1LΔC103 exhibited an effect on inhibiting tumor growth, but the anti-tumoral efficacy of FILIP1LΔC103 was enhanced when combined with DDP compared to the therapy of FILIP1LΔC103 or DDP alone. Data are expressed as Means±SE. **p<0.01 versus untreated group; ^++^*p*<0.01 versus HPEI+FILIP1LΔC103-p group.

To evaluate the tumor suppressor function of HPEI+FILIP1LΔC103-p plus DDP combination treat-ment *in vivo*, a human ovarian cancer intraperitoneal xenograft model was established. Nude mice in the five groups were treated as aforementioned, and then euthanized at the cessation of animal experiment. As shown in Figure 2, the group that undergone therapy with HPEI+FILIP1LΔC103-p or DDP alone exhibited effective inhibition of tumor growth (mean tumor weight, 0.51±0.25 g, and 0.38±0.11 g, respectively). The growth of tumor observed in the two groups were inhibited significantly when compared against the two control group treated with 5% GS and HPEI+E-p (mean tumor weight, 1.71±0.31 g, and 1.61±0.19 g, respectively, p<0.01). However, the combination therapy group (mean tumor weight, 0.13±0.04g) exhibited an augmented effect on tumor suppression when compared with the two control group treated with HPEI+FILIP1LΔC103-p and DDP alone (P<0.01, Figure 2B and 2C).

Intraperitoneal tumor nodules of various range of sizes were disseminated predominantly in the pelvic area as well as inferior to the liver in the 5% GS and HPEI+E-p groups (Figure 2A). Four mice in the 5% GS group was found to have ascites, and three mice in HPEI+E-p treated group, which included hemorrhagic ascites in two mice and one mice from 5% GS, and HPEI+E-p group, respectively (Table 1). No mice from the HPEI+FILIP1LΔC103-p, DDP, and HPEI+FILIP1LΔC103-p plus DDP groups had ascites, and the tumor nodules from mice in these groups were all limited to the pelvic area.

**Table 1:** Characterization of intraperitoneal xenografts of human ovarian cancer in the different treatment group in nude mice

Groups (n=5)	Number of nodules (means ± SD)	Diameter of nodules	Number of hemorrhagic ascites/ascites	Average weight (g, means ± SD)
Untreated	10±3	3 - 14	2/4	1.71±0.31
HPEI+E-p	9±3	2 - 12	1/3	1.61±0.19
HPEI+FILIP1LΔC103	4±1	1 - 5	0/0	0.51±0.25
DDP	4±1	1 - 4	0/0	0.38±0.11
HPEI+FILIP1LΔC103 plus DDP	2±1	0.5 - 2	0/0	0.13±0.04

### Effect of the combination treatment on angiogenesis

**Figure 3:**
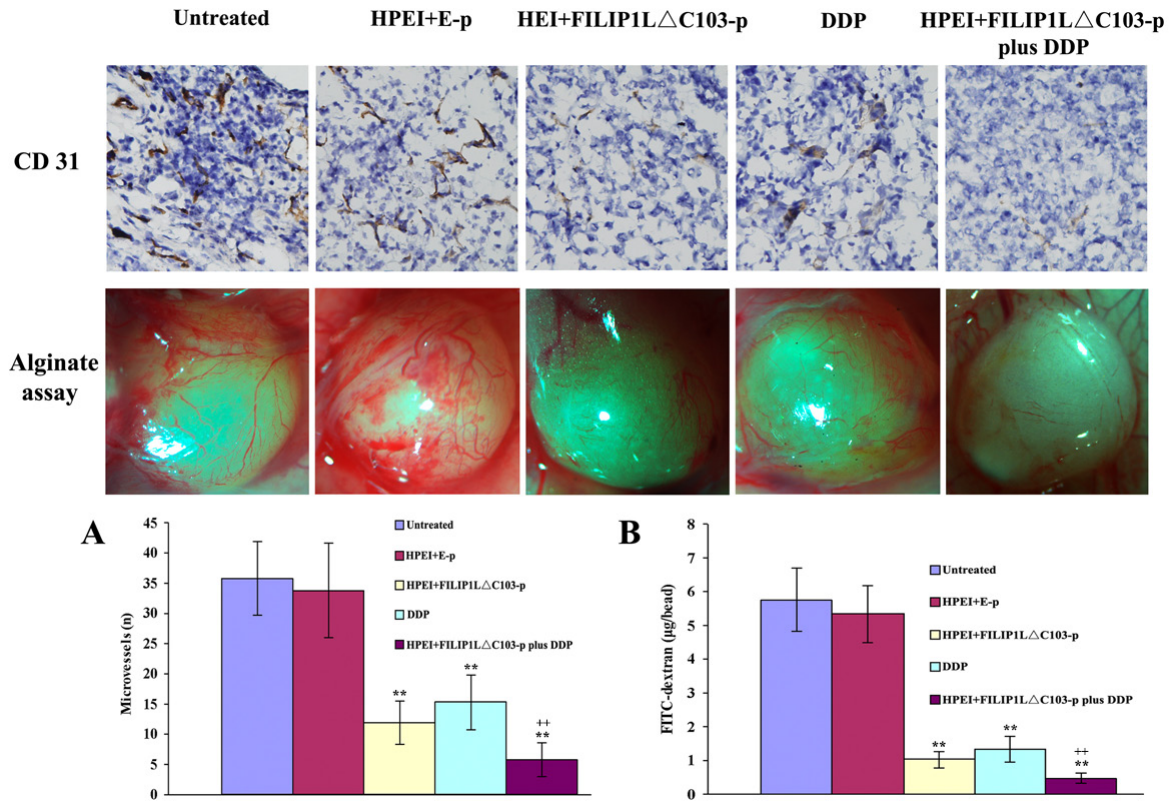
Effect of the combination treatment on angiogenesis Inhibition of angiogenesis was determined by CD31 immunostaining and alginate-encapsulate tumor cells assay *in vivo*. **(A)** CD31 immunostaining revealed that there were sparse microvessels in the tumor tissues of the HPEI+FILIP1LΔC103-p and DDP monotherapy groups, however fewer blood vessels was observed in the combination therapy group compared to controls. **(B)** Vascularization of alginate beads. Mice were treated with 5%GS, HPEI+E-p complexes, HPEI+FILIP1LΔC103-p complexes, DDP or HPEI+FILIP1LΔC103-p plus DDP. Beads were surgically removed and photographed. The suppression of angiogenesis occurred in the HPEI+FILIP1LΔC103-p or DDP monotherapy groups. The function of anti-angiogenesis was more powerful in the combination treatment group compared to sole treatment. Meanwhile, FITC-dextran uptake of beads from the different group revealed the similar result. Data are shown as Means ± SE. **p<0.01 versus untreated group; ^++^*p*<0.01 versus HPEI+FILIP1LΔC103-p group.

To investigate possible mechanisms behind the antitumor effect of HPEI+FILIP1LΔC103-p plus DDP *in vivo*, we will take a further look at its anti-angiogenesis effects. Anti-vascularization is broadly considered to be a significant mechanism for tumor treatment. Frozen sections of tumor were stained by antibodies of CD31 to examine the anti-vascular effect of HPEI+FILIP1LΔC103-p plus DDP. Neovascularization in different sections from the five group tumor tissues was determined by microvessel density (MVD). Analysis of CD31 immunochemical staining indicated that a significant reduction of MVD was observed in the tumor tissues from groups treated with HPEI+FILIP1LΔC103-p or DDP alone compared with untreated or HPEI+E-p group (p<0.01, Figure 3A), and the most significant reduction of MVD in the tumor sections was observed from mice received the HPEI+FILIP1LΔC103-p plus DDP combination therapy compared with HPEI+FILIP1LΔC103-p or DDP alone (P<0.01).

Furthermore, alginate-encapsulate tumor cell assay was performed to detect the functionality of anti-vascularization. New microvessels in the alginate beads were obviously sparse in the nude mice treated with HPEI+FILIP1LΔC103-p or, but rich and twisted microvessels could be detected in alginate beads from 5% glucose and HPEI+E-p treated groups. New microvessels were markedly few in the alginate beads from HPEI+FILIP1LΔC103-p plus DDP group. Moreover, the results of FITC-dextran uptake were consistent with what we observed in the alginate beads from five groups, the levels of FITC-dextran uptake was markedly lower in the nude mice treated with HPEI+FILIP1LΔC103-p plus DDP in comparison with monotherapy groups (P<0.01 vs. HPEI+FILIP1LΔC103-p group, Figure 3B).

### Effect of the combination treatment on cell apoptosis

**Figure 4:**
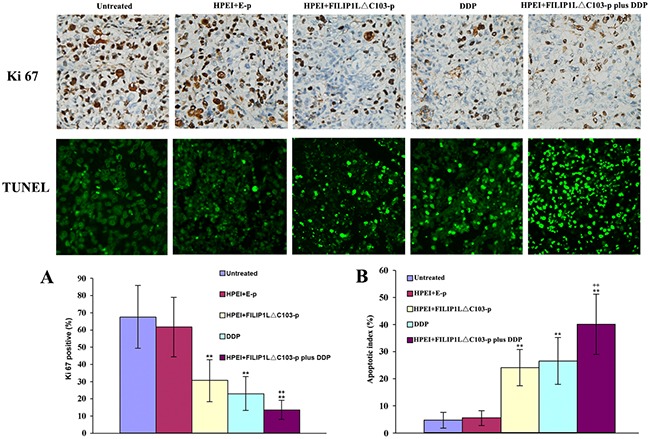
Effect of the combination treatment on cell proliferation and apoptosis *in vivo* **(A)** The significant suppression of cell proliferation was detected in the HPEI+FILIP1LΔC103-p or DDP single treatment groups. Many Ki-67-positive cells were identified in the untreated and HPEI+FILIP1LΔC103-p groups, but only a few positive nuclei occurred in HPEI+FILIP1LΔC103-p plus DDP group. **(B)** Apoptotic cells could be observed in tumors treated with HPEI+FILIP1LΔC103-p or DDP alone. Combination therapy obviously increased the percentage of apoptotic cells versus controls. Data are shown as Means ± SE. **p<0.01 versus untreated group; ^++^*p*<0.01 versus HPEI+FILIP1LΔC103-p group.

Apoptotic cells in ovarian cancer tissues were detected using TUNEL method. Cell nuclei presented as dark green was recorded as positive apoptotic cell nuclei. As shown in Figure 4, obvious cell apoptosis was observed in the tumor from the nude mice undergoing treatment with the FILIP1LΔC103-p or DDP alone when contrasted against 5% GS or E-p. The most substantial cell apoptosis in the tumors was observed from the nude mice in the combination treatment group when compared against FILIP1LΔC103-p or DDP alone (P<0.01 vs. FILIP1LΔC103-p).

### Effect of the combination treatment on cell proliferation

We evaluated tumor cell proliferation using Ki-67 immunostaining. A significant reduction of Ki-67 positive cells was identified in tumor sections from the nude mice undergoing treatment with HPEI+FILIP1LΔC103-p or DDP alone when contrasted against 5%GS or E-p, whereas the most substantial reduction of Ki-67 positive cells occurred in tumor sections from the nude mice undergoing treatment with HPEI+FILIP1LΔC103-p plus DDP in comparison with FILIP1LΔC103-p or DDP alone (Figure 4, P<0.01 vs. FILIP1LΔC103-p). Meanwhile, no substantial discrepancy in tumor cell proliferation was observed between the 5% GS and HPEI+E-p groups.

### Histologic analysis

**Figure 5:**
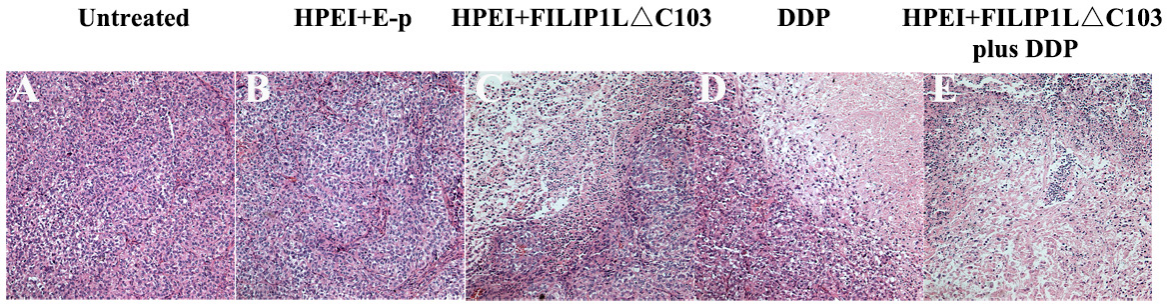
Histological analysis of tumors Tumor tissues from the untreated group **(A)** and HPEI+E-p group **(B)** had large areas of confluent tumor cells with little or no tumor tissue necrosis. Tumors with distinct necrosis were shown in HPEI+FILIP1LΔC103-p **(C)**, DDP **(D)**, and HPEI+FILIP1LΔC103-p plus DDP groups **(E)**, respectively. Representative sections of tumor tissue in each treatment group were depicted at ×200 magnification.

Histologically, the FILIP1LΔC103-p treated tumors revealed vast areas of necrosis or apoptosis (Figure 5D), which were similar to DDP-treated tumors in size and morphology (Figure 5C). Little or no tumor tissue apoptosis or necrosis was discovered in tumor tissues from the untreated group and E-p group (Figure 5A and 5B). Analysis of the degree of tumor tissue apoptosis/necrosis showed that combination treatment of FILIP1LΔC103-p plus DDP was apparently more effective, exhibiting a significant increasement of tumor necrosis and apoptosis related to monotherapy of FILIP1LΔC103-p or DDP (Figure 5E).

### Assessment of toxicity

**Figure 6:**
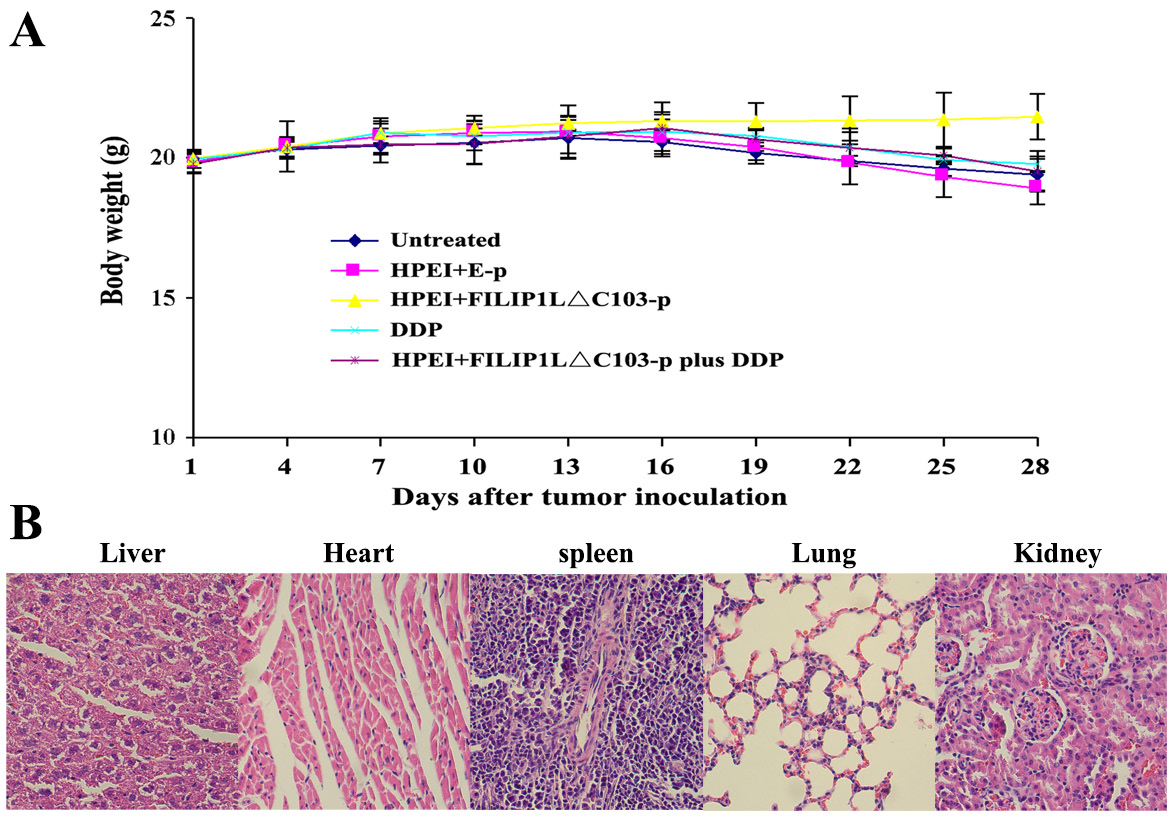
Lack of obvious toxicity-dependent weight loss in treated nude mice **(A)** There were no significant differences in body weight among the five groups (*p*>0. 05), although minor weight loss occurred in the DDP and combination therapy groups. Data are expressed as Means±SD. **(B)** HE histological staining of liver, heart, spleen, lung, and kidney from mice treated HPEI+FILIP1LΔC103-p plus DDP did not demonstrate any significant pathologic differences compared to the control groups. Representative sections of important organs tissue in the combination treatment group were photomicrographed at ×400 magnification.

The body weight of mice, used to evaluate physical status, anorexia or cachexia, was recorded every third day. There was only a insignificant decrease but no substantial differences in the body weight of the nude mice receiving DDP treatment; likewise, no statistically significant differences were found in the body weight of all the nude mice (P>0.05, Figure 6). Moreover, no signs of cumulative adverse results, such as ruffling of skin and abnormal behavior were observed. Liver, lung, heart, spleen, and kidney H&E histological staining assay revealed that no statistically significant differences could be seen among these five groups.

## DISCUSSION

Filamin A interacting protein 1-like (FILIP1L) was determined as a novel angiogenesis activity regulator. Up-regulated expression of FILIP1L could lead to induction of tumor cell apoptosis and tumor cell proliferation inhibition, FILIP1LΔC103 (COOH terminal truncation mutant 1-790 of FILIP1L) is more effective than wild-type FILIP1L in mediating these activities [1]. We have demonstrated that overexpression of FILIP1LΔC103 could efficiently inhibit human ovarian cancer intraperitoneal xenograft growthin our previous study [2]. FILIP1L was first discovered to be expressed in human normal ovarian epithelial cells, but invariably nonexistent in ovarian cancer cell lines [18]. A recent study had shown that down-regulated expression of FILIP1L is related to the invasive phenotype in ovarian tumor, indicating that FILIP1L has the potential to be a biomarker for invasive potential and a target for novel ovarian cancer therapy [3].

Most of the ovarian cancer patients are diagnosed at the advanced clinical stage and have a poor prognosis, and chemotherapy combined with cytoreductive surgery is the main treatment for ovarian cancer. Cisplatin (DDP) is a first-line chemotherapeutic agents in the management of ovarian carcinoma patients. However, the clinical response to DDP is unsatisfactory in treating ovarian cancer due to its dose-dependent toxicity and drug resistance. The systemic side-effects and toxicity associated with high dose often cause patient intolerance. In addition, the gynecologic oncologists have reached a plateau with these conventional chemotherapeutic agents. To address these issues, targeted therapy was introduced. However, numerous preclinical and clinical trials have demonstrated that sole targeted therapy cannot achieve a desired therapeutic effect. In such clinical settings, a dual agent approach instead of a mono agent potentially could be a more advantageous alternative with the aim of reducing toxicity and increasing antitumor efficiency. It is more logical to form effective alternative combinatorial therapies combining chemotherapy agents with functional novel targeted gene therapies at present stage.

This study is a novel endeavor to investigate a prospective effective strategy of combining targeted gene FILIP1LΔC103 with low-dose DDP in the ovarian carcinoma therapy. The most important findings of the study are that combination treatment of FILIP1LΔC103 and low-dose DDP revealed synergistic antitumor effect that either of them alone could not achieve, including inhibition of angiogenesis, suppression of tumor growth and enhancement of tumor cell apoptosis.

The mechanisms of enhanced anticancer effect remain to be fully elucidated. The enhanced anticancer effect of combination treatment *in vivo* may be ascribed to reduced angiogenesis, effective inhibition of proliferation of ovarian cancer cell and increased induction of apoptosis of ovarian cancer cell. These speculations are supported by the present findings. Firstly, the inhibition of angiogenesis was seen in the tumor sections of the nude mice undergoing treatment with FILIP1LΔC103 or DDP alone. In the current study, CD31 immunohistochemical staining and alginate-encapsulated tumor cell assay *in vivo* revealed that evident angiogenesis inhibition was observed in FILIP1LΔC103 or DDP treatment compared against the control groups. However, the lowest count of newborn microvessels was found in the mice group receiving combination treatment amongst the five groups. FILIP1LΔC103 has been identified to be a key regulator of tumor angiogenesis and FILIP1LΔC103 has the potential to be an anti-angiogenic mediator in the ovarian carcinoma treatment. DDP has been shown to exert anti-cancer activity in many different tumor cell lines through tumor cell apoptosis induction by damaging the DNA [19, 20]. There was also a study showing that DDP could inhibit the endothelial cell proliferation through suppression of DNA synthesis [21]. It seems that the pro-apoptotic and anti-proliferative efficacy of DDP to ovarian cancer cells are enhanced when FILIP1LΔC103 was re-expressed in ovarian carcinoma cells. The expression of FILIP1LΔC103 and cytotoxicity of DDP are in synergy with each other on the aspect of inhibiting angiogenesis. Secondly, a significant increase of apoptotic cells was detected in the combination treatment group compared against the monotherapy group (FILIP1LΔC103 or DDP alone). Due to reduced microvessel density and vascular perfusion as a result of inhibition of neovascularization, cancer cells do not receive adequate nourishment during their re-growth after chemotherapeutic insults. Moreover, increased vascular permeability, which results in increased exposure of cancer cells to chemotherapeutic drugs, could be caused by an impaired endothelium, and the pro-apoptotic efficacy of DDP are thus strengthened. In this study TUNEL immunohistochemical assay demonstrated that FILIP1LΔC103 or DDP could result in significant tumor cell proliferation inhibition and tumor cell apoptosis induction, while a more apparent effect was observed in the combination treatment group. Nevertheless, the fundamental molecular mechanism of the inhibition of tumor neovascularization, and thus the ovarian tumor cell apoptosis, need to be fully elucidated in the future.

Tumor necrosis could theoretically account for augmented cell killing [22]. In this study, an analysis of the degree of tumor tissue necrosis demonstrated that the efficacy of killing of FILIP1LΔC103 plus DDP was clearly more significant, suggesting that an apparent increase in tumor necrosis was associated with a single element treatment of FILIP1LΔC103 or DDP (Figure 5). DDP also appears to kill the targeted cells in a caspase-independent manner, apart from inducing cell apoptosis [23]. A study has shown that the capability of DDP to kill transformed fibroblastic cells and MCF-7 cells is directly related to the density of cells, probably as a result of the creation of intercellular gap junctions [24]. Thus, the possibility that the enhancive killing efficacy, that possibly might result in an increased tumor necrosis, might also include the enhanced penetration of DDP by FILIP1LΔC103 into intercellular gap junctions of cancer cannot be excluded.

It is well known that the gene therapy success is strongly dependent its delivery system. As a new non-viral gene carrier, HPEI nanogels hold many advantages over the other non-viral gene delivery system, such as low cytotoxicity, biodegradability, high transfection efficiency [12]. HPEI nanogels could effectively deliver the FILIP1LΔC103 gene into SKOV3 cells, and expression of FILIP1LΔC103 *in vitro* and *in vivo* could be found at the mRNA and protein level. In our previous study, a toxicity evaluation revealed that HPEI+FILIP1LDC103-p complex treatment was safe and without discernable toxicity at the dosage used [2]. Similarly, no overt cytotoxicity and systemic toxic effects were observed in the current study. FILIP1LΔC103 delivered by HPEI nanogels displayed a superb tolerance in the intraperitoneal treatment procedure.

The success of the combination treatment in the current study is based on drug administration routine and the dosing/scheduling strategy which were adopted for the therapy. Ovarian cancer nodules were mainly located in the abdominal and pelvic cavity, therefore a better drug concentration in the tumor region might not be obtained through systemic administration. Compared to systemic delivery, intraperitoneal delivery of HPEI+FILIP1LΔC103 or DDP may refrain from systemic toxicity and first pass effect [25], and maintain a high local drug concentration in the tumor region where it was locally applied. *in vitro* results showed that 72 hours after plasmid transfection, the FILIP1LΔC103 expression attenuation was distinct, therefore FILIP1LΔC103-p was given to the mice every third day at a dosage of 5 μg. On the basis of the sole consideration of toxicity, administration of DDP was conducted in a similar fashion. It was administered to the mice at the dosage of 3mg/kg weekly instead of at maximum tolerated dosage (9 mg/kg/week) [26]. In this study, the increased efficacy without the overt toxicity implied the efficiency of the dosing/scheduling strategy and intraperitoneal administration routine.

In summary, our data suggest that FILIP1LΔC103, as a potential angiogenesis inhibitor, has anti-tumor activity when overexpressed in cancer cells through inhibiting angiogenesis, tumor cell apoptosis induction and diminishing tumor cell proliferation. The anti-tumoral efficacy of FILIP1LΔC103 is enhanced when combined with low-dose DDP without an apparent increase in toxicity. Biodegradable cationic HPEI nanogels could serve as a competent and safe gene carrier, without perceivable toxicity at the dosage that was used. Thus, HPEI nanogels loading FILIP1LΔC103 amalgamated with DDP might become a novel and prospective therapeutic strategy in the ovarian carcinoma treatment.

## MATERIALS AND METHODS

### Cell culture

Human umbilical vein endothelial cells (HUVECs) were isolated from human umbilical vein vascular wall (the experiment procedure was approved by the Ethics Committee of West China Second Hospital of Sichuan University, Chengdu, China. The fresh human umbilical cords were obtained from normal pregnant women without any known disease undergoing vaginal delivery at the West China Second Hospital, and the written informed consent was also obtained) as described in a previous study below [13]. In a brief summary, human umbilical vein vascular wall was digested with collagenase IV at 37°C for 10 minutes, and the homogenate was centrifuged at 750 G for 10 minutes. Cells were suspended and seeded on fibronectin-coated plates, and cultured in Earles’ salts medium supplemented with 10% fetal calf serum (FCS). The human ovarian serous cystadenocarcinoma cells line SKOV3 was obtained from American Type Culture Collection (ATCC, Manassas, VA) and cultured in RPMI-1640 medium (GIBCO, Carlsbad, CA) supplemented with 2mM L-glutamine, 10% fetal bovine serum (FBS), 100 units/ml penicillin G and 100 μg/ml streptomycin and passaged on reaching 70-80% confluence at a split ratio of 1:3 using trypsin, then Cells were incubated in a humidified atmosphere containing 5% CO2 at 37°C.

### RNA extraction and reverse transcription polymerase chain reaction (RT-PCR)

Total RNA was extracted from HUVECs using Trizol reagent (Invitrogen) following the manufacturer's instructions. Based on the sequence of cDNA of mFILIP1LΔC103, the RNA was subjected to RT-PCR for amplification of its encoding region, using a One-Step-RNA-PCR kit (AMV, TaKaRa) with upstream primer 5’-*CGC***GGATCC**AAGATGGTGGTGGATGAACAGCA-3’ and downstream primer 5’-*CCG***CTCGAG**TCACGCATGCTTGGCACTGATTT-3’. The incorporated 5-BamHI and 3-XhoI restriction sites were highlighted in bold typeface while protective base in italics. Reverse transcription from RNA to cDNA was performed at 50°C, 30 minutes, 94°C, 2 minutes (1 cycle); The PCR parameters used in the current study were: 94°C, 30 seconds; 55°C, 30 seconds; and 72°C, 3 minutes (30 cycles). Three μl of amplified product were electrophoresed on 1% agarose gel.

### Construction of recombinant plasmid encoding FILIP1LΔC103

pVAX1 plasmid (Invitrogen, San Diego, CA) encoding mutant-type FILIP1L (FILIP1LΔC103) named FILIP1LΔC103-p was constructed in our laboratory as described in a previous study [2]. The pVAX1 Plasmid without FILIP1LΔC103 (named E-p) was used as an empty-vector control. Large-scale plasmid DNA was purified using an EndoFree Plasmid Giga kit (Qiagen). The DNA was dissolved in sterile endotoxin-free water and adjusted eventually to 1.0 mg/ml, and then stored at -20°C for the future use.

### Preparation of HPEI and transfection of plasmid

The biodegradable cationic HPEI nanogels were synthesized at the State Key Laboratory of Biotherapy and Cancer Center as described in a previous study [12]. The synthesized HPEI nanogels were adjusted to a final concentration of 1.0 mg/ml and stored at 4°C.

The SKOV3 cell transfection was carried out using HPEI nanogels. In our previous study, the optimal efficiency of transfection could be obtained when the plasmid/HPEI ratio was 1:10 *in vitro*. 2.0×10^5^ SKOV3 ovarian cancer cells were grown on six-well plates in RPMI-1640 medium and cultured for twenty-four hours. 20μg HPEI and 2μg plasmid (FILIP1LΔC103-p or E-p) were diluted in 1ml RPMI-1640 medium without antibiotic and serum, respectively, and then mixed at a ratio of 10:1. The complexes were transfected to cells with 50-60% confluence. Six hours later, the medium was replaced by the 2ml RPMI-1640 medium. After forty-eight hours of incubation, the cells and supernatants were collected for a further assay.

### Western blot

Western blot was performed to monitor the expression of protein FILIP1LΔC103. In brief, cells or tumor tissues were lysed in modified radio-immunoprecipitation assay (RIPA) lysis buffer containing protease inhibitor Phenylmethanesulfonyl fluoride (PMSF) and then centrifuged at 1200 rpm for 30 minutes. 20 μg of the total protein were separated on 10% sodium dodecyl sulfate-polyacrylamide gel electrophoresis (SDS-PAGE) gel and subsequently transferred to polyvinylidene fluoride (PVDF) membrane. The membrane was then incubated with rabbit anti-FILIP1L polyclonal antibody (Santa Cruz Biotechnology, Santa Cruz, CA, USA) overnight at 4°C followed by horseradish peroxidase(HSP)-conjugated anti-rabbit secondary antibody. The bands were visualized with the enhanced chemiluminescence (ECL) detection system (Pierce Biotech Inc., Rockford, IL, USA).

### Animal tumor model and treatment

All animal experimental procedures were in compliance with the National Institutes of Health Guide for the Care and Use of Laboratory Animals and approved by the Institutional Animal Care and Treatment Committee of the Sichuan University (Chengdu, People's Republic of China). Six to eight-weeks-old Female athymic BALB/c nude mice were housed in autoclaved microisolator cages. 5×10^6^ SKOV3 cells in 100 μl of RPMI 1640 were injected subcutaneously into the backs of nude mice using a 25 gauge needle, and then mice were euthanized when the diameter of tumors reached up to 1cm, approximately forty days after injection. Tumors were collected, minced into small particles with a diameter of less than 1mm. RPMI 1640 without FBS was added to mix with the tumor particles, and the mixture reached up to a final volume of 12.5 ml [14]. Subsequently, 25 nude mice were injected intraperitoneally with 0.5 ml of the mixture using a 14-guage needle, and randomly assigned to one of the following five groups (5mice per group): (a) untreated group, 100 μl of 5% glucose solution (5%GS). (b) HPEI+E-p group, 5 μg E-p/50 μl HPEI in 50 μl of 5% GS. (c) HPEI+ FILIP1LΔC103-p group, 5 μg FILIP1LΔC103-p/50μl HPEI in 50 μl of 5% GS solution. (d) DDP group, 3 mg/kg (Qinu Pharmacy Corporation, China). (e) combination group (HPEI+ FILIP1LΔC103-p plus DDP group), combination therapy containing treatment of 5 μg FILIP1LΔC103-p/50 μg HPEI complexes for 12 times and DDP (3 mg/kg) for 3 times (volume=100 μl). We had tested various dosages of DDP and demonstrated that the dose of 3 mg/kg/week was safe and effective for mice in our laboratory. To mimic ‘metronomic’ chemotherapy, that is relatively frequent administrations of relatively low doses of chemotherapy, the intraperitoneal administration for DDP was given weekly for a total of 3 times and the other treatments were carried out every three days for 12 times. Side-effects of the treatment, such as weight loss, appetite, and change of behavior of the mice were observed as well. The mice were euthanized and dissected intraperitoneal tumors were weighed 3 days after the last injection.

### Alginate-encapsulated tumor cell assay

To explore inhibition of angiogenesis, an alginate-encapsulation assay was done. Briefly, SKOV3 cells were resuspended in a 1.5% solution of alginate (Sigma) and added dropwise into a solution of 250 mM CaCl2, an alginate bead was formed containing 1×10^5^ cells. Four beads were then implanted subcutaneously in the back of the nude mice. Ten mice were then grouped and treated as aforementioned. Treatment was initiated on the same day of implanting beads. After two weeks, the mice were injected intravenously with 0.2 ml of a 50 mg/kg FITC-dextran (Sigma) solution. Alginate beads were photographed after being exposed surgically, then rapidly removed twenty minutes after FITC-dextran injection. The uptake of FITC-dextran was measured as described in a previous study [15].

### Immunohistochemical analysis

Tissue sections of 3-5 μm from tumors were fixed in 4% neutral buffered formalin, embedded in paraffin, treated with 3% H_2_O_2_ for thirty minutes to dispose of the endogenous peroxidase activity, and followed by blockage with 5% normal serum for 30 minutes. Subsequently, the sections were incubated with primary antibodies for two hours in a humidified chamber at 37°C. After three washes with Phosphate Buffered Saline (PBS, each for five minutes), sections were incubated with HRP-conjugated secondary antibodies for thirty minutes at 37°C.

### TUNEL assay

Terminal deoxynucleotidyl transferase-mediated nick end-labeling (TUNEL) assay was performed to detect the apoptotic cells in tumor tissues (Promega, Madison, WI, USA). Cell nuclei displaying dark green fluorescence in tissues were recorded as TUNEL-positive cells. The apoptosis index was calculated by analyzing the average percentages of positive apoptotic cells in ten random fields from different sections using a ×400 magnification.

### Quantification of angiogenic micro-vessel density

Micro-vessel density (MVD) was determined by examining the areas with vascular hot spots as described in a previous study [16]. Five vascular tumor areas without necrosis were selected and viewed at a high magnification of ×200. Vessels within the high-power fields of these areas, with a clearly defined lumen or well defined linear vessel shape, were taken into account for as microvessel, and an average of the five values was recorded for each sample.

### Histological analysis

Tumors fixed in 10% neutral buffered formalin were embedded in paraffin and sliced into 5 μm sections for staining with hematoxylin and eosin (H&E) as described in a previous study [17] and examined by a pathologist in a blind manner.

### Observation of toxicity

Relevant side effects of treatment, such as diarrhea, loss of weight, cachexia, anorexia, appetite, toxic death, skin ulceration, or behavior were monitored consecutively to evaluate the toxicity of treatment. In addition, sections of organs e.g. lung, kidney, spleen, liver, heart were stained with H&E, and then observed by a pathologist in a blind manner.

### Statistical analysis

All experimental results were recorded as mean ± standard deviation (SD). One-way of variance (AVOVA) was performed with SPSS software (version 19.0 for Windows) to calculate statistical significance among experimental data from different groups. Difference was defined as significant at P<0.05.
